# Practical and conceptual issues of clinical trial registration for Brazilian researchers: Aspectos práticos e conceituais do registro de ensaios clínicos para pesquisadores brasileiros

**DOI:** 10.1590/1516-3180.2014.00441803

**Published:** 2015-08-28

**Authors:** Carolina Gomes Freitas, Thomas Fernando Coelho Pesavento, Maurício Reis Pedrosa, Rachel Riera, Maria Regina Torloni

**Affiliations:** I BPharm. Master’s Student in the Postgraduate Evidence-Based Healthcare Program, Universidade Federal de São Paulo (UNIFESP), and Research Assistant at the Brazilian Cochrane Center, São Paulo, Brazil.; II PT. Physiotherapist. Master’s Student in the Postgraduate Evidence-Based Healthcare Program, Universidade Federal de São Paulo (UNIFESP), and Research Assistant at the Brazilian Cochrane Center, São Paulo, Brazil.; III BSc. Biochemist. Master’s Student in the Postgraduate Evidence-Based Healthcare Program, Universidade Federal de São Paulo (UNIFESP), and Research Assistant at the Brazilian Cochrane Center, São Paulo, Brazil.; IV MD, MSc, PhD. Professor in the Postgraduate Evidence-Based Healthcare Program, Department of Evidence-Based Medicine, Universidade Federal de São Paulo, and Researcher at the Brazilian Cochrane Center, São Paulo, Brazil.; V MD, MSc, PhD. Professor in the Postgraduate Evidence-Based Healthcare Program, Department of Evidence-Based Medicine, Universidade Federal de São Paulo, Director of the Brazilian Cochrane Center, and Researcher at the Brazilian Cochrane Center, São Paulo, Brazil.

**Keywords:** Clinical trials as topic, Database [publication type], Information systems, Publication bias, Brazil

## Abstract

**CONTEXT AND OBJECTIVE:**

Clinical trial registration is a prerequisite for publication in respected scientific journals. Recent Brazilian regulations also require registration of some clinical trials in the Brazilian Clinical Trials Registry (ReBEC) but there is little information available about practical issues involved in the registration process. This article discusses the importance of clinical trial registration and the practical issues involved in this process.

**DESIGN AND SETTING:**

Descriptive study conducted by researchers within a postgraduate program at a public university in São Paulo, Brazil.

**METHODS:**

Information was obtained from clinical trial registry platforms, article reference lists and websites (last search: September 2014) on the following topics: definition of a clinical trial, history, purpose and importance of registry platforms, the information that should be registered and the registration process.

**RESULTS:**

Clinical trial registration aims to avoid publication bias and is required by Brazilian journals indexed in LILACS and SciELO and by journals affiliated to the International Committee of Medical Journal Editors (ICMJE). Recent Brazilian regulations require that all clinical trials (phases I to IV) involving new drugs to be marketed in this country must be registered in ReBEC. The pros and cons of using different clinical trial registration platforms are discussed.

**CONCLUSIONS:**

Clinical trial registration is important and various mechanisms to enforce its implementation now exist. Researchers should take into account national regulations and publication requirements when choosing the platform on which they will register their trial.

## RESUMO

**CONTEXTO E OBJETIVO:**

O registro dos ensaios clínicos é pré-requisito para publicação em revistas científicas de prestígio. Recentes mecanismos regulatórios brasileiros também exigem o registro de determinados ensaios clínicos na plataforma nacional (Registro Brasileiro de Ensaios Clínicos, ReBEC). Porém há pouca informação disponível sobre questões práticas envolvidas no processo de registro. Este trabalho discute a importância do registro de ensaios clínicos e aspectos práticos envolvidos nesse processo.

**DESENHO DE ESTUDO E LOCAL:**

Estudo descritivo realizado por pesquisadores de um programa de pós-graduação em uma universidade pública em São Paulo, Brasil.

MÉTODOS: Informações foram obtidas em plataformas de registro de ensaios clínicos, referências dos artigos encontrados e *websites* (última busca: setembro, 2014) sobre os seguintes temas: definição de ensaio clínico, história, objetivo e importância das plataformas de registro, quais informações devem ser registradas e o processo de registro.

**RESULTADOS:**

O registro de ensaios clínicos visa evitar viés de publicação e é exigido por revistas brasileiras disponíveis no LILACS, SciELO e revistas afiliadas ao Comitê Internacional de Editores de Revistas Médicas (ICMJE). Recentes normas brasileiras exigem que todos os ensaios clínicos (fases I a IV) envolvendo novos medicamentos no país sejam registrados no ReBEC. São discutidos os prós e contras da utilização de diferentes bases de registro de ensaios clínicos.

**CONCLUSÃO:**

O registro de ensaios clínicos é importante e atualmente existem vários mecanismos que obrigam seu uso. Quando forem escolher em qual plataforma registrar seu ensaio clínico, os pesquisadores devem levar em consideração quais são as normas nacionais e as exigências para publicação.

## INTRODUCTION

Registration of clinical trials has received increasing attention over the last few years. Discussions have progressed from theoretical issues, such as the importance of establishing trial registration platforms^1^ and the creation of these registries, to regulatory issues involving enforcement of trial registration, such as through publication and legal restraints.^2^^,^^3^^,^^4^^,^^5^^,^^6^ Many published papers on the history of trial registries are available at the Ottawa Group website,^7^ including the 2004 Ottawa Statement, which presents the main principles for development of these registries.^8^

Over the last 14 years, the Brazilian government has recognized the strategic importance of scientific research for the country and has created mechanisms and structures to administer and encourage research.^9^^,^^10^^,^^11^ In consonance with this plan, the Brazilian Clinical Trials Registry (ReBEC) has been created to register and provide information on clinical trials conducted in Brazil.^12^

Previous published papers have analyzed the importance of trial registries in general and of the ReBEC platform in particular for Brazilian researchers.^12^^,^^13^^,^^14^ However, to the best of our knowledge, no previous papers have assessed the recent changes in the regulatory scenario of trial registries or provided any practical guidance for Brazilian researchers on how to register their trials. This gap motivated us to write this paper, which presents theoretical and practical issues relating to trial registration in Brazil.

## OBJECTIVES

This study aimed to inform Brazilian researchers about the history and importance of clinical trial registration and to offer practical advice on how to use the ReBEC platform.

## METHODS

This was a descriptive study. We searched MEDLINE (via PubMed), SciELO and LILACS from inception to September 30, 2014, for information on clinical trial registration, using the plain text “randomized controlled trial”, OR “registry databases” OR “randomized controlled trial registration” and their corresponding Brazilian terms “Ensaio Clínico Controlado Aleatório”, “Bases de Registros” and “Registro de Ensaio Clínico Randomizado”, respectively. We complemented the search by screening the reference lists of articles selected for full text reading and by searching the websites of organizations with registry platforms or involved in the regulatory processes of this initiative, such as the International Committee of Medical Journal Editors (ICMJE),^15^ the Cochrane Collaboration,^16^ the World Health Organization (WHO),^17^ the Ottawa group^1^ and the Brazilian Registry of Clinical Trials (ReBEC).^18^

From these sources, we extracted information on 1) the definition of a trial, 2) the history and importance of trial registries and 3) details on where, when and how Brazilian researchers can register their trials.

## RESULTS

### What is a trial?

A trial is a study that prospectively assigns human beings or groups of human beings to one health-related intervention or to a series of such interventions, in order to assess the effects of the interventions on health.^15^^,^^17^ A trial, also known as an interventional study, can test many different types of medical interventions used to modify health outcomes, such as drugs, biological products, surgical procedures, devices, behavioral treatments, preventive measures and healthcare protocols, among others.^19^

### History and importance of trial registries

Before conducting a trial, researchers usually write a protocol that provides a brief contextual description of the problem or disease that will be investigated, the specific research question, the objectives of their study and some details on the methods that will be used, such as the participant selection criteria, a description of the intervention and the exact outcomes that will be assessed, as well as statistical issues such as sample size calculation and how the data will be analyzed.^20^ Although this study protocol is naturally written by all investigators prior to actually beginning their study, until recently it was not mandatory to publish or register this protocol anywhere.

Registration of a trial protocol is an ethical pledge to ensure transparency in the execution and publication of studies.^3^^,^^6^^,^^8^^,^^12^^,^^13^^,^^15^^,^^21^ WHO highlights that trial registries can also be sources of evidence on the efficacy and safety of health interventions.^22^

The main reasons for clinical trial registration are:

- To avoid publication bias.^3^^,^^8^^,^^12^^,^^13^^,^^14^^,^^15^ Registration of a trial protocol, in theory, ensures that the results of that study will be published, regardless of whether the findings were beneficial, harmful, inconclusive or even inefficacious.
- To avoid selective reporting.^3^^,^^8^^,^^12^^,^^13^^,^^14^^,^^15^ Registration of all essential details about outcomes that will be analyzed in the study protocol will preclude authors from selecting which outcome data will later be published.
- To honor the ethical participant-investigator covenant.^3^^,^^8^^,^^12^^,^^13^^,^^14^^,^^15^ In theory, registration of a trial ensures that both the methods and the results of the study will be published. Through publishing the study, the investigators will fulfill their ethical responsibility to the participants, because the data gathered in the study will be used to advance scientific knowledge.

Brazilian investigators have additional unique motivations for registration of their clinical trials, such as:

- To disseminate their work, thereby enabling greater visibility for Brazilian researchers.^6^^,^^12^
- To avoid language bias.^6^^,^^12^^,^^23^ Non-English speaking researchers tend to only publish studies with positive results in English-language journals, thus limiting the dissemination of unfavorable results. Registration of the study protocol promotes dissemination of all trials, regardless of their findings since most clinical trial registries, included ReBEC bring both native language and English protocol versions.

Although discussions about mandatory registration of trial protocols date back to the 1960s,^1^ this idea only gained momentum in the following century. In 2000, two large trial registries, ClinicalTrials.gov and Current Controlled Trials (ISRCTN), were created. Five years later, the ICMJE issued a recommendation on the use of these registries and many top medical journals, such as the Lancet, the British Medical Journal and BioMed Central journals, started to require prospective trial registration as a prerequisite for considering manuscripts for publication.^24^^,^^25^ In 2007, this recommendation was also adopted by all Brazilian journals indexed in the Latin American and Caribbean Health Sciences Literature (LILACS) database and by those available through the Scientific Electronic Library Online (SciELO).^3^

Some years later, the Brazilian platform for the registration of clinical trials (Registro Brasileiro de Ensaios Clínicos, ReBEC) was created. ReBEC is administered by Fundação Oswaldo Cruz (FIOCRUZ), in association with the Brazilian Ministry of Health, the Pan-American Health Organization (PAHO) and the Latin American and Caribbean Center on Health Sciences Information (BIREME). This platform allows free open-access registration of any trial that started recruiting participants after January 2010. In 2008 and 2012, two Brazilian regulatory laws (RDC 39/2008 and RDC 36/2012) were passed.^4^^,^^5^ Since 2012, all clinical trials (phases I to IV) involving new drugs to be marketed in Brazil must be registered on this trial platform.^4^^,^^5^

### Where, when and how to register a trial

In 2007, the World Health Organization created a network of clinical trial registries called the International Clinical Trials Registry Platform (ICTRP).^26^ This secondary platform combines 16 of the most popular primary trial registry platforms on a single webpage (apps.who.int/trialsearch).^27^

To meet the publication requirements of the ICMJE, the trial protocol must be registered in one of the primary registries of the ICTRP network.^15^ Trial authors in any country can chose freely where to register their protocols in one of several primary trial registries available worldwide. Examples of these registries include the Brazilian platform (ReBEC),^18^ the Australian New Zealand Clinical Trials Registry (ANZCTR),^28^ the Chinese Clinical Trial Registry (ChiCTR),^29^ the Cuban Public Registry of Clinical Trials (RPCEC),^30^ the Pan-African Clinical Trial Registry (PACTR),^31^ the EU Clinical Trials Register (EU-CTR)^32^ and the International Standard Randomized Controlled Trial Number Register (ISRCTN.org),^33^ among others. However, some platforms, such as ISRCTN, charge fees for registration of trial protocols while others, like the Pan-African Clinical Trials Registry and the EU Clinical Trials Registry, only register trials conducted locally. Once registered in any of the affiliated registries, the text of the trial protocol cannot be deleted and in some of them (e.g. ClinicalTrials.gov),^12^^,^^17^ protocol amendments also become public.^34^

Before registering the trial protocol, the authors need to have obtained approval for their study through local or national ethics committees. The real recruitment of participants should only begin after the trial registration has been officially completed and is available online, since registration is not accepted after that point.^6^^,^^14^ Clinical trials not registered from inception will face difficulties in the publishing process, since they will not be accepted by ICMJE-affiliated and probably many other journals.

The key elements needed for registering trials on these platforms are very simple and are usually part of any study protocol. Box 1 describes key data required for registering a trial in the WHO network (ICTRP) of affiliated platforms.^35^

**Box 1.**
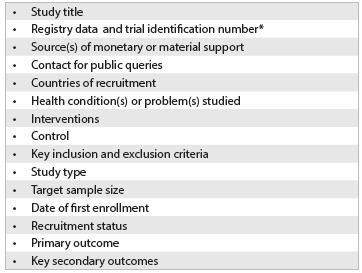
Main information required by World Health Organization (WHO) for registration of a trial in any International Clinical Trials Registry Platform (ICTRP) affiliated registries.^35^

*generated by the system.

### Practical advice on registration in ReBEC

Registration of a trial on the Brazilian platform follows steps that are similar to those of other trial registries. Details and answers to frequently asked questions are provided on the ReBEC website.^6^

**Figure 1.**
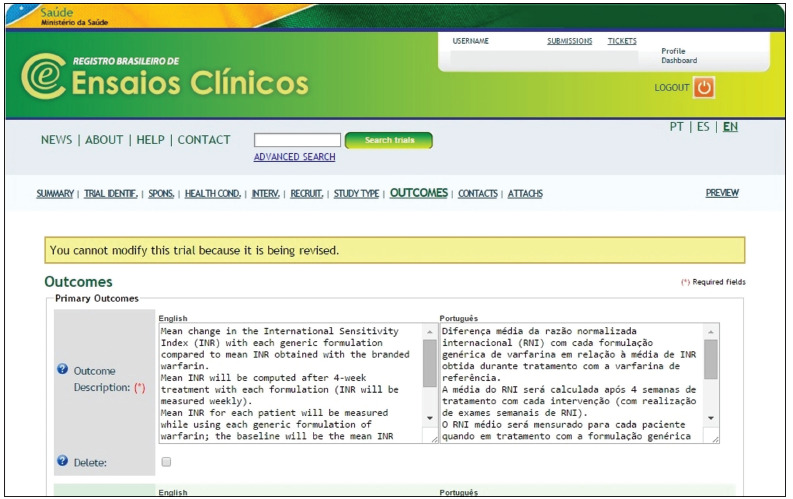
Screen shot of part of the Outcomes section of a Registro Brasileiro de Ensaios Clínicos (ReBEC) trial submission form. Outcomes are described in free-text fields.

We should nevertheless point out some inconvenient features of ReBEC, such as the need to fill out many free-text fields (Figure 1). Another problem is that the instructions on how to fill out the fields are not very clear. As a result, the process of trial registration is prone to errors and this will lead to several bouts of revision dialogue between ReBEC and the applicant.

One of the present authors (CGF) compared the registration process of a trial already registered in ClinicalTrials.gov (identifier NCT02017197) with the registration process in ReBEC. While it took approximately one week between submission and publication of the trial register in ClinicalTrials.gov in 2013, the same study protocol, which was submitted to ReBEC in April 2014, has not yet been published online as of February 20, 2015. The reason for this delay may be that the ClinicalTrials.gov registration form, unlike that of ReBEC, has an interface with many closed questions, which probably speeds up the registration process. Not only that, since 2014 and until now (April 15) the homepage of ReBEC displays a message of staff shortage, which leads us to think that the registration process will be even slower.

## DISCUSSION

This study describes the history and importance of clinical trial registry platforms and provides useful information for Brazilian researchers on where and how to register their trials. This pragmatic approach is one of the strengths of the study, since it responds to the needs of researchers who are usually not interested in extensive scientific discussions about clinical trial registration or specific characteristics of some registries but want practical information on this essential topic.^3^^,^^8^^,^^12^^,^^13^^,^^14^^,^^21^ This need influenced our search strategy, which was not restricted to scientific articles, but included searches on relevant websites, along with a practical exercise on the Brazilian platform.

Registration of clinical trials emerged from scientific and ethical concerns on research transparency and is now going through a process of scientific and legal regulation aimed at enforcing its implementation.

We recommend that before selecting a specific platform for trial registration, researchers should pay attention to national regulations and laws, which may vary depending on their geographical setting. For Brazilian researchers, or foreign investigators conducting clinical trials in Brazil, the ReBEC platform is an option. ReBEC meets the requirements for publication both in Brazilian and in foreign journals and, in some cases (e.g. for registration of new drugs on the Brazilian market), it is mandatory to register trials on this platform. However, ReBEC has several shortcomings because of its format, and this can considerably delay the process of trial registration. We think that many of the problems identified in ReBEC are probably due to the fact that this registry and the regulations on trial registration are relatively new in Brazil.

However, recent information regarding staff shortages at ReBEC means that improvement in its performance will not happen over the short term, which is highly detrimental to the reputation of the platform and also an impediment for companies that aim to register new drugs in Brazil.

## CONCLUSIONS

Clinical trial registration has over 50 years of history and has received increasing attention over the last decade. Brazil has had a national trial registry (ReBEC) since 2010 and regulations have been created to encourage its use. However, this platform has some drawbacks. Researchers should take into account national laws and publication requirements when choosing the platform on which they will register their trial.
